# Association of Corpus Callosum Development With Fetal Growth Restriction and Maternal Preeclampsia or Gestational Hypertension

**DOI:** 10.1001/jamanetworkopen.2022.26696

**Published:** 2022-08-15

**Authors:** Weizeng Zheng, Xiaodan Zhang, Yan Feng, Bingqing Liu, Jiajun Zhu, Yu Zou, Jiale Qin, Baohua Li

**Affiliations:** 1 Department of Radiology, Women’s Hospital, Zhejiang University School of Medicine, Hangzhou, China; 2 Department of Obstetrics, Women’s Hospital, Zhejiang University School of Medicine, Hangzhou, China; 3 Department of Women’s Health, Women’s Hospital, Zhejiang University School of Medicine, Hangzhou, China; 4 Department of Neonatology, Women’s Hospital, Zhejiang University School of Medicine, Hangzhou, China; 5 Department of Ultrasound, Women’s Hospital, Zhejiang University School of Medicine, Hangzhou, China; 6 Women’s Reproductive Health Key Laboratory of Zhejiang Province, Women’s Hospital, Zhejiang University School of Medicine, Hangzhou, China

## Abstract

**Importance:**

It remains unknown whether neurodevelopmental impairments are directly associated with the structural development of the brain in offspring with fetal growth restriction (FGR) and mothers with preeclampsia (PE) or gestational hypertension (GH).

**Objectives:**

To assess whether fetal corpus callosum (CC) development differed among pregnancies with PE or GH with FGR, pregnancies with PE or GH without FGR, and normotensive pregnancies, particularly the severity of maternal disease and FGR, and to identify the association between adverse perinatal outcomes and structural development of the CC in fetuses with FGR in pregnancies with PE or GH.

**Design, Setting, and Participants:**

This retrospective matched case-control study was conducted between January 1, 2014, and January 31, 2021, at Women’s Hospital, Zhejiang University School of Medicine in Hangzhou, China. The participant group included cases of singleton pregnancies with PE or GH with FGR; the control groups included cases with PG or GH without FGR and cases with paired normotensive pregnancy.

**Exposures:**

Maternal PE or GH and FGR.

**Main Outcomes and Measures:**

The length, thickness, total area, subdivision areas, and apparent diffusion coefficient (ADC) values of fetal CC were measured on magnetic resonance imaging (MRI) and analyzed. The association between adverse perinatal outcomes and structural development of CC was further investigated.

**Results:**

A total of 56 pregnant individuals with singleton pregnancies and PE or GH and fetuses with FGR were enrolled (maternal median [IQR] age, 29.0 [26.0-34.0] years; mean [SD] gestational age at MRI, 33.6 [2.5] weeks). Significant patterns of decreased median (IQR) fetal CC length (0.4284 [0.4079-0.4470] mm vs 0.4614 [0.4461-0.4944] mm, *P* < .001, vs 0.4591 [0.4310-0.4927] mm, *P* < .001) and mean (SD) CC total area (1.0779 [0.1931] mm^2^ vs 1.1896 [0.1803] mm^2^, *P* = .001, vs 1.1438 [0.1935] mm^2^, *P* = .02), adjusted for the cephalic index, was observed in cases of PE or GH with FGR compared with cases without FGR and cases with normotensive pregnancy. The splenium region of fetal CC also exhibited the distinct alterations in macrostructural development (with FGR: 0.3149 [0.0697] mm^2^ vs without FGR: 0.3727 [0.0698] mm^2^, *P* < .001, vs normotensive pregnancies: 0.3565 [0.0763] mm^2^, *P* < .001) and microstructural development (median [IQR] ADC values: 1.47 [1.38-1.57] × 10^−3^ mm^2^/s vs 1.57 [1.53-1.63] × 10^−3^ mm^2^/s, *P* = .009, vs 1.63 [1.50-1.70] × 10^−3^ mm^2^/s, *P* < .001) in all groups. Furthermore, significant associations were found between structural abnormality of the splenium region and adverse perinatal outcomes in the PE or GH with FGR group (mean [SD] ADC value: 1.40 [0.07] × 10^−3^ mm^2^/s; *P* = .04).

**Conclusions and Relevance:**

Results of this study suggest that, in fetuses with FGR in pregnancies with PE or GH, decreased structural development of the CC, predominantly the splenium region, may be significantly associated with a higher risk of adverse perinatal outcomes and may be regarded as an MRI-based biomarker for better prenatal counseling and early management decisions.

## Key Points

**Question:**

What is the association between maternal preeclampsia (PE) or gestational hypertension (GH) complicated by fetal growth restriction (FGR) and structural development of the fetal corpus callosum (CC)?

**Findings:**

In this case-control study of 56 cases of singleton pregnancies with PE or GH with FGR, reduced structural development of the CC, predominantly the splenium region, was associated with an increase in adverse perinatal outcomes.

**Meaning:**

The findings of this study suggest that fetuses with FGR who have mothers with PE or GH may experience abnormal structural development of the CC.

## Teaser

This case-control study evaluates the neurodevelopmental and perinatal outcomes in offspring of pregnant individuals with preeclampsia or gestational hypertension.

## Introduction

Preeclampsia (PE) or gestational hypertension (GH) is a common medical problem that is associated with a range of maternal and fetal complications, including kidney dysfunction, hepatic injury, pulmonary edema, eclampsia, fetal growth restriction (FGR), and stillbirth.^1,2^ Evidence from epidemiologic studies shows that PE or GH affects 2% to 10% of all pregnancies worldwide and is associated with some neurodevelopmental disorders in offspring, especially complicated by FGR.^1,3,4^

Fetal growth restriction is a pathological condition in which a fetus does not reach its expected growth potential. Although the use of FGR as a diagnostic criterion for PE remains controversial, the adverse maternal and perinatal outcomes are more common in patients with PE or GH that is complicated by FGR.^5,6,7^ Currently, ultrasonography is used as the primary tool to evaluate fetal growth capability and perform FGR diagnosis, and fetal magnetic resonance imaging (MRI) may be used as an adjunct to ultrasonography to assess the associated brain anomalies of fetuses with FGR. Clinical MRI studies of fetuses with FGR have shown fetal brain injuries in utero that lead to reduced cerebral volume,^8^ alterations in cortical morphometry,^9^ and the induction of microstructural changes.^10,11^ Nevertheless, little is known of whether PE or GH plays a role in inducing distinct structural abnormalities in the brain of fetuses with FGR as shown on MRI.

The development of the corpus callosum (CC) is a complex process involving the establishment and maturation of gray and white matter connections between the cerebral hemispheres. The CC has been proposed as a sensitive indicator of intrauterine brain development and maturation.^10,12^ A prospective study has reported that fetuses small for gestational age show a substantial macrostructural reduction of the CC and smaller subdivision areas, compared with fetuses appropriate for gestational age.^10^ Furthermore, diffusion-weighted imaging (DWI) provides apparent diffusion coefficient (ADC) values as a measure of water diffusion and is associated with cellular density and proliferative indexes; this finding provides a foundation for understanding microstructural changes in fetal neurodevelopmental disorders.^11^ A prospective multicenter cohort study using DWI in the setting of FGR detected lower ADC values in the frontal white matter territory of fetuses and showed that this reduction in ADC was associated with adverse perinatal outcomes.^13^ To our knowledge, no published study has examined the association of maternal PE or GH complicated by FGR with macrostructural and microstructural development of the fetal CC.

In the present study, we detected distinct macrostructural and microstructural alterations in fetal CC between PE or GH with or without FGR and normotensive pregnancy. The aim of this study was 2-fold: (1) to ascertain whether fetal CC development differed among pregnancies with PE or GH with FGR, pregnancies with PE or GH without FGR, and normotensive pregnancies, particularly the severity of maternal disease and FGR, and (2) to assess whether there was an association between adverse perinatal outcomes and structural development of the CC in fetuses with FGR in pregnancies with PE or GH.

## Methods

A retrospective case-control study was conducted at Women’s Hospital, Zhejiang University School of Medicine in Hangzhou, China, between January 1, 2014, and January 31, 2021. The study was approved by the institutional review board of Women’s Hospital, Zhejiang University School of Medicine. All participants provided written informed consent. We followed the Strengthening the Reporting of Observational Studies in Epidemiology (STROBE) reporting guideline.

### Participants and Clinical Data Collection

We identified singleton cases of PE or GH (following the 2020 American College of Obstetricians and Gynecologists criteria^5^) whose ultrasonographic findings were suggestive of FGR complication, defined as an abdominal circumference below the 10th centile with a pulsatility index greater than 95th centile in either the umbilical artery or uterine artery.^14^ Subsequently, a fetal MRI was performed within 1 week. We confirmed cases of PE or GH with or without FGR using birth weight percentile.^15^ Fetal growth restriction was defined as birth weight less than the 10th centile, whereas severe FGR was defined as birth weight below the third centile.^16^

The control groups were (1) cases of PE or GH without FGR and (2) normotensive pregnancy deemed low risk because of isolated polyhydramnios with normal karyotypes and morphological features, maternal placental disorders, and benign gynecologic tumors on MRI. Excluded criteria are discussed in the eMethods in the Supplement.

A flowchart of the selection of study participants from the enrolled pregnant patients is shown in eFigure 1 in the Supplement. According to a 1:1 ratio, we matched the control groups for gestational age, maternal age, and neonatal sex. Participants self-reported race and ethnicity information, which was routinely collected. The race and ethnicity identified by individuals included Han and minority groups. The final cohort consisted of 3 groups: cases of PE or GH with FGR, cases of PE or GH without FGR, and cases of normotensive pregnancy.

Clinical data were retrieved from the medical record databases of Women’s Hospital, Zhejiang University School of Medicine (Table 1). We collated adverse perinatal outcomes among neonates with FGR of patients with a PE or GH diagnosis. Adverse perinatal outcomes were a composite measure of any of the following: neonatal death, hypoxic-ischemic encephalopathy, neonatal necrotizing enterocolitis, and pulmonary hemorrhage.^13^

**Table 1.** Maternal and Perinatal Clinical Characteristics Among the Study Groups

Characteristic	Cases of PE or GH		Cases of normotensive pregnancy (n = 56)
	Fetus with FGR (n = 56)	Fetus without FGR (n = 56)	
Maternal age, median (IQR), y	29.0 (26.0-34.0)	30.0 (27.3-32.8)	30.5 (28.0-33.0)
Higher educational level, No. (%)^a^	37 (66)	44 (79)	42 (75)
Smoking, No. (%)	0	1 (2)	0
No. of routine antenatal care visits, median (IQR)^b^^,^^c^	6.00 (4.00-8.75)	10.00 (7.25-12.00)	9.00 (8.00-11.00)
Pregestational BMI, median (IQR)^d^	21.32 (20.01-23.82)	22.69 (20.86-25.36)	22.01 (20.31-23.31)
Nulliparous, No. (%)	38 (68)	43 (77)	34 (61)
Fertility treatment, No. (%)	3 (5)	7 (13)	3 (5)
Gestational diabetes, No. (%)	14 (25)	15 (27)	NA
PE, No. (%)	34 (61)	26 (46)	NA
Antihypertensive treatment, No. (%)	41 (73)	40 (71)	NA
Cesarean delivery, No. (%)^c^	45 (80)	35 (63)	24 (43)
Emergency cesarean delivery, No. (%)^c^	23 (41)	17 (30)	9 (16)
GA at US, mean (SD), wk	33.3 (2.7)	33.3 (2.7)	33.3 (2.8)
GA at MRI, mean (SD), wk	33.6 (2.5)	33.6 (2.5)	33.6 (2.6)
GA at PE or GH diagnosis, mean (SD), wk	31.5 (4.5)	31.5 (5.6)	NA
GA at delivery, median (IQR), wk^b^^,^^c^^,^^d^	36.0 (34.0-38.3)	38.5 (37.3-39.5)	39.0 (38.5-40.0)
Umbilical arterial S/D pressure ratio at US before delivery, median (IQR)^b^^,^^c^	2.70 (2.50-3.18)	2.50 (2.33-2.70)	2.40 (2.10-2.70)
Birth weight, mean (SD), g^b^^,^^c^^,^^d^	1923.21 (632.42)	3065.87 (453.94)	3225.63 (362.10)
Male to female ratio	0.75	0.75	0.60
Apgar score at 1 min, median (IQR)^b^^,^^c^^,^^d^	10.00 (9.00-10.00)	10.00 (10.00-10.00)	10.00 (10.00-10.00)
Apgar score at 5 min, median (IQR)^b^^,^^c^	10.00 (10.00-10.00)	10.00 (10.00-10.00)	10.00 (10.00-10.00)
NICU admission rate, No. (%)^b^^,^^c^	16 (29)	2 (4)	0
Length of NICU stay, median (IQR), d^b^^,^^c^	0.0 (0.00-4.25)	0	0

Abbreviations: BMI, body mass index (calculated as weight in kilograms divided by height in meters squared); FGR, fetal growth restriction; GA, gestational age; GH, gestational hypertension; MRI, magnetic resonance imaging; NA, not applicable; NICU, neonatal intensive care unit; PE, preeclampsia; S/D, systolic/diastolic; US, ultrasonography.

^a^ Higher educational level was defined as college degree or higher.

^b^ *P* value for PE or GH with FGR vs PE or GH without FGR was *P* < .05.

^c^ *P* value for PE or GH with FGR vs normotensive pregnancy was *P* < .05.

^d^ *P* value for PE or GH without FGR vs normotensive pregnancy was *P* < .05.

### MRI Acquisition Protocol and MRI Analysis

We evaluated the macrostructural and microstructural changes of the fetal CC between the 3 study groups using T2-weighted anatomical images and DWI on MRI. The macrostructural and microstructural measurements of MRI included linear, thickness, and area measurements of the fetal CC as well as ADC measurements according to standard protocol (eFigure 2 in the Supplement). To correct for differences in head size, we considered the cephalic index (CI) by measuring the biparietal diameter (BPD) and occipitofrontal diameter (OFD): CI = BPD/OFD × 100.^17^ All of the length, thickness, and area measurements of the CC were adjusted by the CI. The eMethods in the Supplement provides specific methodological details.

### Statistical Analysis

The reliability between measurements, which were acquired by 2 of us (W.Z. and X.Z.) who were blinded to participants’ group membership, was assessed by intraclass correlation coefficients and their 95% CIs (eTable 1 in the Supplement). Data were expressed as number (percentage), median (IQR), or mean (SD), as appropriate. Continuous data were tested for normality using the Kolmogorov-Smirnov test. For comparisons of matched groups, we used the paired, 2-tailed *t* test or the Wilcoxon matched-pairs test according to continuous data distribution. For intragroup comparisons in cases of PE or GH with FGR, we performed unpaired, 2-tailed *t* test or Mann-Whitney test for continuous data.

For all comparisons, 2-tailed *P* < .05 was considered significant. All tests were performed using IBM SPSS Statistics for Windows, version 21.0 (IBM Corp).

## Results

### Clinical Characteristics

The cohort comprised 56 cases of singleton pregnancies with PE or GH with FGR (median [IQR] maternal age, 29.0 [26.0-34.0] years; mean [SD] gestational age at MRI, 33.6 [2.5] weeks), which were subdivided into PE with FGR (34 [61%]) and GH with FGR (22 [39%]). Of these cases, 14 (25%) had less severe FGR and 42 (75%) had severe FGR. Following the paired principle, we included in the control groups 56 cases of PE or GH without FGR and 56 cases of normotensive pregnancy.

Maternal characteristics and perinatal outcomes are shown in Table 1. Maternal baseline characteristics did not differ significantly among the 3 groups, except for the median (IQR) number of routine antenatal care visits, which was significantly lower in cases of PE or GH with FGR (6.00 [4.00-8.75] visits) compared with the control cases (without FGR: 10.00 [7.25-12.00] visits, *P* < .001; normotensive pregnancies: 9.00 [8.00-11.00] visits, *P* < .001). There were no statistical differences in the gestational age at ultrasonography or MRI, although gestational age at delivery was earlier, and the median (IQR) umbilical arterial systolic/diastolic ratio at ultrasonography before delivery was worse in cases of PE or GH with FGR (2.70 [2.50-3.18]) than in control cases (without FGR: 2.50 [2.33-2.70], *P* < .001; normotensive pregnancies: 2.40 [2.10-2.70], *P* < .001). Furthermore, fetuses in the PE or GH with FGR group, when compared with fetuses in the control groups without FGR and normotensive pregnancies, had a significantly lower mean (SD) birth weight (1923.21 [632.42] g vs 3065.870 [453.94] g, *P* < .001; vs 3225.63 [362.10] g, *P* < .001), lower median (IQR) Apgar score at 1 minute (10.00 [9.00-10.00] vs 10.00 [10.00-10.00], *P* = .01; vs 10.00 [10.00-10.00], *P* < .001) and lower median (IQR) Apgar score at 5 minutes (10.00 [10.00-10.00] vs 10.00 [10.00-10.00], *P* = .03; vs 10.00 [10.00-10.00], *P* = .01), higher rates of neonatal intensive care unit admission (16 [29%] vs 2 [4%], *P* < .001; vs 0, *P* < .001), and a longer median (IQR) length of neonatal intensive care unit stay (0 [0-4.25] days vs 0 days, *P* < .001; vs 0 days, *P* < .001).

### Macrostructural Changes of the Fetal CC

A comparison of macrostructural changes of the fetal CC in the PE or GH with FGR group and the control groups is shown in Table 2. Results showed that fetuses with FGR in cases of PE or GH had reduced mean (SD) BPD (7.98 [0.70] cm vs 8.37 [0.56] cm and 8.54 [0.60] cm) and median (IQR) OFD (9.38 [8.97-9.76] cm vs 9.95 [9.64-10.43] cm and 10.10 [9.39-10.52] cm) compared with fetuses in the control cases. To eliminate the disturbance of reduced BPD and OFD, we calculated the CIs and used them as adjusted ratios. With regards to the linear and area measurements (adjusted for the CI) of the fetal CC, the PE or GH with FGR group, compared with the control groups without FGR and normotensive pregnancies, showed a significant pattern of a shorter median (IQR) CC length (0.4284 [0.4079-0.4470] mm vs 0.4614 [0.4461-0.4944] mm, *P* < .001; vs 0.4591 [0.4310-0.4927] mm, *P* < .001) and a smaller mean (SD) CC total area (1.0779 [0.1931] mm^2^ vs 1.1896 [0.1803] mm^2^, *P* = .001; vs 1.1438 [0.1935] mm^2^, *P* = .02). Furthermore, in analysis of the different subdivision areas of the fetal CC, we observed a linear tendency for a significantly reduced mean (SD) splenium area (adjusted for the CI) across the 3 groups (with FGR: 0.3149 [0.0697] mm^2^; vs without FGR: 0.3727 [0.0698] mm^2^, *P* < .001; vs normotensive pregnancies: 0.3565 [0.0763] mm^2^, *P* < .001).

**Table 2.** Macrostructural Analysis of Fetal CC Among the Study Groups

Parameter of fetal brain	Mean (SD)		
	Cases of PE or GH		Cases of normotensive pregnancy (n = 56)
	Fetus with FGR (n = 56)	Fetus without FGR (n = 56)	
BPD at MRI, cm^a^^,^^b^^,^^c^	7.98 (0.70)	8.37 (0.56)	8.54 (0.60)
OFD at MRI, median (IQR), cm^a^^,^^b^	9.38 (8.97-9.76)	9.95 (9.64-10.43)	10.10 (9.39-10.52)
CI^a^^,^^c^^,^^d^	86.20 (5.16)	84.02 (4.22)	85.71 (3.63)
CC length/CI, median (IQR), mm^a^^,^^b^^,^^d^	0.4284 (0.4079-0.4470)	0.4614 (0.4461-0.4944)	0.4591 (0.4310-0.4927)
Genu thickness/CI, median (IQR), mm^a^^,^^c^^,^^d^	0.0187 (0.0150-0.0233)	0.0217 (0.0174-0.0241)	0.0190 (0.0165-0.0228)
Body thickness/CI, mm^d^	0.0146 (0.0027)	0.0145 (0.0028)	0.0148 (0.0029)
Splenium thickness/CI, median (IQR), mm^d^	0.0263 (0.0210-0.0343)	0.0289 (0.0247-0.0368)	0.0316 (0.0227-0.0356)
Anterior third/CI, mm^2^^a^^,^^d^	0.4078 (0.0739)	0.4371 (0.0685)	0.4269 (0.0606)
Anterior midbody/CI, mm^2^^d^	0.1355 (0.0296)	0.1418 (0.0283)	0.1359 (0.0317)
Posterior midbody/CI, median (IQR), mm^2^^a^^,^^d^	0.1214 (0.1028-0.1451)	0.1336 (0.1180-0.1554)	0.1249 (0.1068-0.1463)
Isthmus/CI, mm^2^^d^	0.0944 (0.0204)	0.1027 (0.0170)	0.0962 (0.0238)
Splenium/CI, mm^2^^a^^,^^b^^,^^d^	0.3149 (0.0697)	0.3727 (0.0698)	0.3565 (0.0763)
Total area/CI, mm^2^^a^^,^^b^^,^^d^	1.0779 (0.1931)	1.1896 (0.1803)	1.1438 (0.1935)

Abbreviations: BPD, biparietal diameter; CC, corpus callosum; CI, cephalic index; FGR, fetal growth restriction; GH, gestational hypertension; MRI, magnetic resonance imaging; OFD, occipitofrontal diameter; PE, preeclampsia.

^a^ *P* value for PE or GH with FGR vs PE or GH without FGR was *P* < .05.

^b^ *P* value for PE or GH with FGR vs normotensive pregnancy was *P* < .05.

^c^ *P* value for PE or GH without FGR vs normotensive pregnancy was *P* < .05.

^d^ CI = BPD/OFD × 100.

The macrostructural differences of the fetal CC in the PE or GH with FGR group were compared according to the severity of maternal and fetal disease. As shown in eTable 2 in the Supplement, a shorter CC length and a smaller splenium area were detected in fetuses with severe FGR compared with fetuses with less severe FGR; however, this phenomenon was not observed in patients in the PE-FGR and GH-FGR groups (eTable 3 in the Supplement).

### Microstructural Changes of the Fetal CC

We acquired the DWI sequences of 40 fetuses in the 56 cases of PE or GH with FGR, and we analyzed the microstructural development of the fetal CC. On axial DWI, ADC values for the anterior third and splenium region were measured (Table 3). Compared with fetuses in the control groups (without FGR and normotensive pregnancies), fetuses in the PE or GH with FGR group had significantly lower median (IQR) ADC values for the anterior third region (1.49 [1.35-1.59] × 10^−3^ mm^2^/s vs 1.59 [1.49-1.68] × 10^−3^ mm^2^/s, *P* = .005; vs 1.58 [1.48-1.69] × 10^−3^ mm^2^/s, *P* = .003) and splenium region (1.47 [1.38-1.57] × 10^−3^ mm^2^/s vs 1.57 [1.53-1.63] × 10^−3^ mm^2^/s, *P* = .009; vs 1.63 [1.50-1.70] × 10^−3^ mm^2^/s, *P* < .001).

**Table 3.** Microstructural Analysis of Fetal CC Among the Study Groups

Parameters of CC DWI	Cases of PE or GH		Cases of normotensive pregnancy (n = 40)
	Fetus with FGR (n = 40)	Fetus without FGR (n = 40)	
ADC of anterior third, median (IQR), ×10^−3^ mm^2^/s^a^^,^^b^	1.49 (1.35-1.59)	1.59 (1.49-1.68)	1.58 (1.48-1.69)
ADC of splenium, median (IQR), ×10^−3^ mm^2^/s^a^^,^^b^	1.47 (1.38-1.57)	1.57 (1.53-1.63)	1.63 (1.50-1.70)

Abbreviations: ADC, apparent diffusion coefficient; CC, corpus callosum; DWI, diffusion-weighted imaging; FGR, fetal growth restriction; GH, gestational hypertension; PE, preeclampsia.

^a^ *P* value for PE or GH with FGR vs PE or GH without FGR was *P* < .05.

^b^ *P* value for PE or GH with FGR vs normotensive pregnancy was *P* < .05.

In fetuses with severe FGR, the ADC value in the splenium region was decreased by approximately 4% compared with fetuses with less severe FGR, although there was no statistical difference (eTable 4 in the Supplement). In addition, no microstructural differences were identified in the fetal CC when compared between those in the PE with FGR group and the GH with FGR group (eTable 5 in the Supplement).

### Association of Macrostructural and Microstructural Development of the Fetal CC With Adverse Perinatal Outcomes

To analyze the association of the fetal CC structural development with adverse perinatal outcomes in cases of PE or GH with FGR, we compared the macrostructural and microstructural changes between the adverse and favorable perinatal outcomes groups. With regards to the macrostructural analysis of the fetal CC (Table 4), fetuses with adverse, compared with favorable, perinatal outcomes showed a significantly shorter median (IQR) CC length (adjusted for the CI) (0.4178 [0.3990-0.4342] mm vs 0.4325 [0.4112-0.4566] mm; *P* = .04). There was no significant difference in the subdivisions of the fetal CC between the 2 groups, although the mean (SD) splenium area (adjusted for the CI) in the adverse perinatal outcomes group was reduced by 10% when compared with the favorable perinatal outcomes group (0.2887 [0.0559] mm^2^ vs 0.3213 [0.0718] mm^2^; *P* = .17). Furthermore, with regard to the microstructural analysis of the fetal CC (Table 5), mean (SD) ADC values in the splenium region were significantly lower in the adverse perinatal outcomes group than in the favorable perinatal outcomes group (1.40 [0.07] × 10^−3^ mm^2^/s vs 1.50 [0.13] × 10^−3^ mm^2^/s; *P* = .04).

**Table 4.** Association Between Macrostructural Change of Fetal CC and Perinatal Outcomes in Cases of Preeclampsia or Gestational Hypertension With FGR

Parameters of fetal brain	Mean (SD)		*P* value
	Favorable perinatal outcomes (n = 45)	Adverse perinatal outcomes (n = 11)	
BPD at MRI, cm	8.07 (0.71)	7.62 (0.55)	.06
OFD at MRI, cm	9.40 (0.66)	8.72 (0.77)	.004
CI, median (IQR)^a^	86.2140 (82.7795-89.3130)	86.3736 (84.9015-89.8325)	.52
CC length/CI, median (IQR), mm^a^	0.4325 (0.4112-0.4566)	0.4178 (0.3990-0.4342)	.04
Genu thickness/CI, median (IQR), mm^a^	0.0182 (0.0148-0.220)	0.0227 (0.0157-0.0240)	.19
Body thickness/CI, mm^a^	0.0144 (0.0027)	0.0158 (0.0024)	.12
Splenium thickness/CI, mm^a^	0.0280 (0.0077)	0.0297 (0.0104)	.56
Anterior third/CI, mm^2^^a^	0.4143 (0.0757)	0.3814 (0.0623)	.19
Anterior midbody/CI, mm^2^^a^	0.1356 (0.0299)	0.1351 (0.0297)	.96
Posterior midbody/CI, mm^2^^a^	0.1259 (0.0284)	0.1210 (0.0263)	.6
Isthmus/CI, mm^2^^a^	0.0948 (0.0209)	0.0926 (0.0192)	.74
Splenium/CI, mm^2^^a^	0.3213 (0.0718)	0.2887 (0.0559)	.17
Total area/CI, mm^2^^a^	1.0918 (0.1986)	1.0209 (0.1640)	.28

Abbreviations: BPD, biparietal diameter; CC, corpus callosum; CI, cephalic index; FGR, fetal growth restriction; MRI, magnetic resonance imaging; OFD, occipitofrontal diameter.

^a^ CI = BPD/OFD × 100.

**Table 5.** Association Between Microstructural Change of Fetal CC and Perinatal Outcomes in Cases of Preeclampsia or Gestational Hypertension With FGR

Parameters of CC DWI	Favorable perinatal outcomes (n = 32)	Adverse perinatal outcomes (n = 8)	*P* value
ADC of anterior third, mean (SD), ×10^−3^ mm^2^/s	1.50 (0.17)	1.43 (0.07)	.42
ADC of splenium, mean (SD), ×10^−3^ mm^2^/s	1.50 (0.13)	1.40 (0.07)	.04

Abbreviations: ADC, apparent diffusion coefficient; CC, corpus callosum; DWI, diffusion-weighted imaging; FGR, fetal growth restriction.

## Discussion

Preeclampsia or GH can be factors in FGR predominantly by prenatal hypoxia and reduced placental blood flow. In this case-control study, we found a significant reduction in fetal CC length and total area in cases of PE or GH with FGR. The splenium region of the fetal CC showed a distinct decrease in macrostructural and microstructural development on MRI when compared with cases of PE or GH without FGR and cases of normotensive pregnancies. This phenomenon was more obvious in fetuses with severe FGR compared with fetuses with less severe FGR. Furthermore, the data revealed a significant association between macrostructural and microstructural development in the fetal CC (predominantly the splenium region) and advent perinatal outcomes in the PE or GH with FGR group.

Prenatal exposure to maternal PE or GH may be associated with the occurrence of FGR and may increase risks of aberrant fetal brain metabolism, function, and neurological disease. Absolute total intracranial volume was significantly smaller in fetuses with FGR than fetuses with appropriate gestational age.^18^ However, the brain-sparing effect implied that some brain structures may be preferentially predisposed to injury induced by maternal PE or GH.^19^ It is still unknown whether fetuses in cases of PE or GH with FGR exhibit more distinct structural changes in the fetal brain. The CC forms the bridge between the cerebral hemispheres, including the anterior third, anterior midbody, posterior midbody, isthmus, and splenium regions, according to the Witelson subdivision. In the present study, we paid close attention to the CC and its subdivision regions because the CC is considered to be an important marker of white matter maturation and easily identified on fetal MRI. We measured the macrostructural differences in fetuses in the 3 study groups and detected a significantly reduced BPD and OFD in cases of PE or GH with FGR. The CI is associated with gestational age and gives a measure of the fetal head shape.^20^ Previous studies^17,21^ have shown that the differences in fetal head size of fetuses with FGR could be reduced by adjusting for the CI; therefore, in this study, all of the linear and area measurements of the CC were adjusted by the CI. Results showed a much shorter CC length and a smaller CC total area (adjusted for the CI) in cases of PE or GH with FGR. The data suggested that, in the subdivision regions of the fetal CC, a reduction in the splenium area was observed only after adjusting for CI. This finding may reflect a specific developmental alteration in fetuses with FGR in pregnancies with PE or GH, compared with fetuses without FGR in pregnancies with PE or GH and fetuses in normotensive pregnancies. In addition, these macrostructural alterations were observed in fetuses with severe FGR rather than in fetuses with less severe FGR.

Diffusion MRI has been used to evaluate fetal neurodevelopment and has revealed different aspects of gray and white matter microstructural changes in vivo.^22^ The ADC concept has been extremely successful in clinical practice, and ADC value as a measure of water diffusion from Brownian motion has been associated with cellular density and proliferative indexes.^23^ Arthurs et al^11^ demonstrated that, in fetuses with severe FGR, ADC values in the frontal white matter, thalami, and centrum semiovale were significantly lower than in control fetuses. A brain imaging study using diffusion tensor imaging provided the first report of lower fractional anisotropy values from the splenium region in premature infants whose mothers had PE, thus indicating that PE might be a factor in a neurodevelopmental delay of the brain microstructure in the CC, especially in the splenium region.^24^ It is unknown whether or to what extent the microstructural alterations occur in the CC of fetuses with FGR in pregnancies with PE or GH. Using diffusion MRI, we identified reduced ADC values in the anterior third region and the splenium region of the fetal CC in the PE or GH with FGR group when compared with the 2 control groups. Furthermore, the ADC values from the splenium region in fetuses with severe FGR were lower than in fetuses with less severe FGR. We believe that the data provide converging evidence for the proposal that fetuses with FGR in pregnancies with PE or GH showed significant macrostructural decrease and lower ADC value in the splenium region of the fetal CC. This finding suggests that the fetal CC, especially the splenium region, in fetuses with FGR in pregnancies with PE or GH may be regarded as a potential MRI-based biomarker for better prenatal counseling and early management intervention.

The splenium region is the most posterior sector of the CC.^25^ Embryonically, the maturational trajectories of the splenium region in utero coincide with the timing of early-onset PE.^12^ Exposure of infants to PE has been associated with significant retardation in the microstructural development of the white matter in the splenium region.^24^ There was evidence that perinatal composite outcomes were increasingly used to evaluate the neonatal prognosis.^13,26^ In a multicenter prospective study of extremely preterm infants, adverse perinatal outcomes (including necrotizing enterocolitis, infection, and intracranial hemorrhage) were associated with a range of high-risk neurodevelopmental impairment assessed at the 2-year follow-up visit.^27^ Results of the present study demonstrated that fetuses with adverse perinatal outcomes had significantly smaller CC length and lower ADC values in the splenium region, compared with fetuses with favorable perinatal outcomes. It is unclear how the abnormal changes in the splenium region mediate the neurodevelopmental dysfunction and associated mechanisms in fetuses with FGR in pregnancies with PE or GH. A previous study has found that the splenium region is involved in visuospatial information transfer, language, reading and calculation scores, and consciousness.^28^ Another study suggested that splenium injury may be associated with language impairment and visual disturbances during childhood.^28,29^ In 2 studies,^30,31^ the offspring with FGR of patients with PE or GH had lower mean verbal IQ and reduced cognitive function. Consequently, we may speculate that the macrostructural and microstructural alterations in the splenium region could further shed light on the specific cognitive impairment in the offspring with FGR of patients with PE or GH.

### Limitations

This study has some limitations. First, complete standardization of the paired conditions was impossible in the retrospective clinical setting. Second, the anatomical landmarks required to delineate the fetal CC were not precise. To resolve this problem, we used a method that has been widely used to measure the CC structural development on MRI^10,32^; the interrater reliability also suggested good agreement between the segmentation results of 2 raters. Third, we studied the microstructural information from only 2 anatomical areas (the anterior third and the splenium regions) of the fetal CC, mainly because of the inherent limitation of axial DWI sequences. Reconstruction of the microstructure of the fetal CC and its subdivisions in utero is associated with several technical difficulties.^33^ In addition, a systematic review found that fetal brain reference ranges on MRI have low-to-moderate methodological quality, increasing the risk of bias and possibly affecting prenatal diagnosis and counseling.^34^ Fourth, we did not evaluate the associations between postnatal neurodevelopmental outcomes and structural alterations in the fetal CC. Hence, ongoing work by our team includes conducting a prospective study with a larger sample size of individuals with PE or GH, acquiring fetal whole-brain structural imaging and diffusion MRI data, and examining the association between fetal brain alterations and poor neurobehavioral performance in infants.

## Conclusions

Findings from this case-control study suggest that structural development of the fetal CC, mainly the splenium region, may be an MRI-based biomarker to identify fetuses with FGR in pregnancies with PE or GH who are at risk for impaired neurodevelopment in utero; this structural development could play a crucial role in the application of individualized decisions and early management intervention. Future studies, such as a large prospective study, need to examine the changes associated with other sections of the fetal brain and to assess the postnatal neurodevelopment of neonates with FGR in cases of PE or GH.

## Notes

Supplement. **eMethods.** Supplementary Methods (Including **eReferences**) **eFigure 1.** A Flowchart Demonstrating the Selection of Study Subjects From the Enrolled Pregnant Women **eFigure 2.** The Macrostructural and Microstructural Measurements of the Fetal Corpus Callosum **eTable 1.** Intraclass Correlation Coefficients Results of Fetal Brain **eTable 2.** Macrostructural Analysis of Fetal Corpus Callosum Between Less Severe FGR and Severe FGR Groups **eTable 3.** Macrostructural Analysis of Fetal Corpus Callosum Between GH-FGR and PE-FGR Groups **eTable 4.** Microstructural Analysis of Fetal Corpus Callosum Between Less Severe FGR and Severe FGR Groups **eTable 5.** Microstructural Analysis of Fetal Corpus Callosum Between GH-FGR and PE-FGR Groups
