# Supplementary material for: Association of Corpus Callosum Development With Fetal Growth Restriction and Maternal Preeclampsia or Gestational Hypertension
Source: JAMA Netw Open. 2022 Aug 15;5(8):e2226696. doi: 10.1001/jamanetworkopen.2022.26696 (PMC9379741; doi:10.1001/jamanetworkopen.2022.26696)
Supplement: Supplement. — eMethods. Supplementary Methods (Including eReferences) eFigure 1. A Flowchart Demonstrating the Selection of Study Subjects From the Enrolled Pregnant Women eFigure 2. The Macrostructural and Microstructural Measurements of the Fetal Corpus Callosum eTable 1. Intraclass Correlation Coefficients Results of Fetal Brain eTable 2. Macrostructural Analysis of Fetal Corpus Callosum Between Less Severe FGR and Severe FGR Groups eTable 3. Macrostructural Analysis of Fetal Corpus Callosum Between GH-FGR and PE-FGR Groups eTable 4. Microstructural Analysis of Fetal Corpus Callosum Between Less Severe FGR and Severe FGR Groups eTable 5. Microstructural Analysis of Fetal Corpus Callosum Between GH-FGR and PE-FGR Groups [file jamanetwopen-e2226696-s001.pdf]

Supplemental Online Content

Zheng W, Zhang X, Feng Y, et al. Association of the corpus callosum development with fetal growth restriction and maternal preeclampsia or gestational hypertension. *JAMA Netw Open*. 2022;5(8):e2226696. doi:10.1001/jamanetworkopen.2022.26696

eMethods. Supplementary Methods (Including eReferences)

eFigure 1. A Flowchart Demonstrating the Selection of Study Subjects From the Enrolled Pregnant Women

eFigure 2. The Macrostructural and Microstructural Measurements of the Fetal Corpus Callosum

eTable 1. Intraclass Correlation Coefficients Results of Fetal Brain

eTable 2. Macrostructural Analysis of Fetal Corpus Callosum Between Less Severe FGR and Severe FGR Groups

eTable 3. Macrostructural Analysis of Fetal Corpus Callosum Between GH-FGR and PE-FGR Groups

eTable 4. Microstructural Analysis of Fetal Corpus Callosum Between Less Severe FGR and Severe FGR Groups

eTable 5. Microstructural Analysis of Fetal Corpus Callosum Between GH-FGR and PE-FGR Groups

This supplemental material has been provided by the authors to give readers additional information about their work.

eMethods. Supplementary Methods (Including eReferences)

Exposure

Preeclampsia (PE) was defined as new-onset hypertension after 20 weeks with proteinuria (≥ 0.3 g of protein in a 24-hour urine specimen). Gestational hypertension (GH) was defined as elevated blood pressure (systolic blood pressure ≥ 140 mm Hg or a diastolic blood pressure ≥ 90 mm Hg) after 20 weeks of gestation without proteinuria in women with previously normal blood pressure.

Maternal, perinatal, and neonatal clinical data were retrieved from the institution's Medical Record Databases, including maternal age, higher education, smoking history, number of routine antenatal care visits, pregestational body mass index, obstetric history, gestational diabetes mellitus, rate of PE, antihypertensive treatment, mode of delivery (cesarean delivery or vaginal delivery), gestational age (GA) at US or MRI, GA at PE or GH diagnosis, GA at delivery, umbilical arterial S/D ratio before delivery, birth weight, neonatal gender, Apgar score, neonatal intensive care unit (NICU) admission rate, and length of NICU stay. GA was determined on the basis of the last menstrual period and confirmed by ultrasound measurement of crown–rump length in the first trimester of pregnancy.

Adverse perinatal outcomes in the current study included neonatal death (two cases), hypoxic-ischemic encephalopathy (six cases), neonatal necrotizing enterocolitis (two cases), and pulmonary hemorrhage (one case).

Exclusion criteria

We excluded fetuses with a GA below 20 weeks, those with chromosomal or structural anomalies as determined prenatally or at birth, and fetuses with fetal hydrops, intrauterine fetal demise, inconsistent GA documentation, missing ultrasound or clinical data, multiple gestation, and insufficient image quality on MRI. In addition, cases of preeclampsia superimposed on chronic hypertension were considered ineligible for the current study.

MRI acquisition protocol

This study was based on the routine fetal MRI procedure carried out in our hospital. All cases were scanned without sedation following the relevant guidelines for use of medical imaging during pregnancy [1,2]. Fetal brain MRI was performed using a 1.5-Tesla system (Signa HDxt; GE Healthcare Technologies, Milwaukee, WI, USA). 2-dimensional multi-slice T_2-weighted anatomical images were obtained using a single-shot fast spin-echo sequence or fast imaging employing steady-state acquisition sequence, and were acquired with the parameters described previously [3]. Diffusion-weighted imaging was performed using single-shot spin-echo-planar imaging in the axial plane, with the following parameters: 20 images in six non-collinear axis directions, spin-echo-planar factor, 95; shortest repetition time, 3100 ms; echo time, 88 ms; field-of-view, 280–350 mm; matrix, 256 × 256; slice thickness, 4 mm with 0.5-mm gap; acquisition time, 57 s. Diffusion-gradient values were b = 0 and b = 600 mm^2/s. In total, the scanning session took about 12–16 min. Specific absorption rate values of all sequences were between 1.0 and 2.5 W/kg.

Linear and area measurements of corpus callosum

On sagittal T_2WI MRI images *in utero*, the normal corpus callosum (CC) is shown as a low-signal intensity stripe arching between the two hemispheres. Linear measurements of the CC were performed following previously reported methodology (eFigure 2) [4]. All macrostructural images were analyzed by

© 2022 Zheng W et al. *JAMA Network Open.*

two senior radiologists (W.Z., X.Z. with 9 years of fetal MRI experience) using Digital Imaging and Communications in Medicine viewer software (RadiAnt DICOM viewer 2020.2; Medixant, Poznan, Poland). CC length was measured from the most anterior part of the genu to the most posterior part of the splenium tracing a straight rostrocaudal line between the two points, known as the outer-to-outer CC length. CC thickness was measured in its anterior, middle and posterior portions corresponding to the genu, body and splenium thickness, respectively. The thickness of the genu was measured from the most anterior point of the CC to the most anterior point of the inner convexity of the genu; the body thickness was defined as the width of the midcallosum; the splenium thickness is the length of the maximal perpendicular between two parallel lines drawn as tangents to the superior and inferior surfaces of the splenium.

CC morphometry was assessed by measuring the total area and the area of a subdivision in five portions: anterior third, anterior midbody, posterior midbody, isthmus, and splenium region, as described by Witelson et al. [4]. The line of CC length was used as the linear axis to subdivide the CC into anterior and posterior halves; anterior, middle, and posterior thirds; and the posterior one-fifth region. The anterior third region of the CC was defined as the anterior one-third. The anterior midbody region of the CC was defined as the anterior half minus the anterior one-third. The posterior midbody region of the CC was defined as the posterior half minus the posterior one-third. The isthmus region was defined as the posterior one-third minus the posterior one-fifth. The splenium region of the CC was defined as the posterior one-fifth.

Diffusion-weighted imaging analysis: apparent diffusion coefficient (ADC) measurement of the CC

All of the fetal DWI sequences were stored and transferred to the vendor-supplied Advantage Workstation (ADW 4.6 version, GE, USA) for centralized analysis performed separately by two investigators (W.Z., X.Z.). ADC measurements of the CC were based upon regions of interests (ROIs) that were previously reported in the literature [5]. Manual placement of the ROIs was carried out on DWI sequence with the axial plane on internal capsule layer, superior to inferior direction, and referred to the histological and MRI fetal brain atlases. The signal intensity was measured in two ROIs, including the anterior third region (ROI 1) and the splenium region (ROI 2) of the CC. The dimension of each ROI was 15-20 mm^2 at less than 32 weeks of gestation and 20-25 mm^2 between 32 and 40 weeks of gestation. To ensure that the ROIs were representative, the relative standard deviation of all pixels needed to be < 10% of the mean ADC values.

Cephalic index of the fetal brain

In order to correct for differences in head size, cephalic index (CI) was considered by measuring the biparietal diameter (BPD) and occipitofrontal diameter (OFD) [6]. To calculate the CI, we used a previously reported formula: CI = BPD/OFD × 100. All of the length, thickness and area measurements of the CC were adjusted by CI, for group comparison purposes.

Reliability between macrostructural and microstructural measurements of the fetal CC

Reliability between measurements from W.Z. and X.Z. blinded to group membership was assessed by the intra class correlation coefficients (ICCs) (two-way random mixed for absolute agreement) and their 95% confidence intervals (eTable 1). To verify the reliability of linear and area measurement, fourteen fetuses (25%) were randomly selected for each group. To verify the reliability of ADC measurement, ten fetuses (25%) were randomly selected for each group. An ICCs of 1 indicates perfect reproducibility between measurements, while a value of 0 is interpreted as reproducibility that is no better

© 2022 Zheng W et al. *JAMA Network Open.*

or worse than that expected by chance. Reproducibility was defined as poor (ICCs < 0.500), moderate (ICCs = 0.500–0.750), good (ICCs = 0.750-0.900), and excellent (ICCs > 0.900).

eReferences

1. Committee Opinion No. 723: Guidelines for Diagnostic Imaging During Pregnancy and Lactation. *Obstet Gynecol.* 2017;130(4):e210-e216. doi:10.1097/AOG.0000000000002355
2. Prayer D, Malinger G, Brugger PC, et al. ISUOG Practice Guidelines: performance of fetal magnetic resonance imaging. *Ultrasound Obstet Gynecol.* 2017;49(5):671-680. doi: 10.1002/uog.17412
3. Shi W, Yan G, Li Y, et al. Fetal brain age estimation and anomaly detection using attention-based deep ensembles with uncertainty. *Neuroimage.* 2020;223:117316. doi:10.1016/j.neuroimage.2020.117316
4. Witelson SF. Hand and sex differences in the isthmus and genu of the human corpus callosum. A postmortem morphological study. *Brain.* 1989;112 (Pt 3):799-835. doi:10.1093/brain/112.3.799
5. Zhang B, Chen D, Qing Z, et al. Growth trajectories and cluster features of the human fetal brain estimated by signal intensity from sBTFE sequence in utero MRI. 2019;1(2):63-73. doi:10.1007/s42058-019-00012-3
6. Paules C, Miranda J, Policiano C, et al. Fetal neurosonography detects differences in cortical development and corpus callosum in late-onset small fetuses. *Ultrasound Obstet Gynecol.* 2021;58(1):42-47. doi:10.1002/uog.23592

© 2022 Zheng W et al. *JAMA Network Open.*

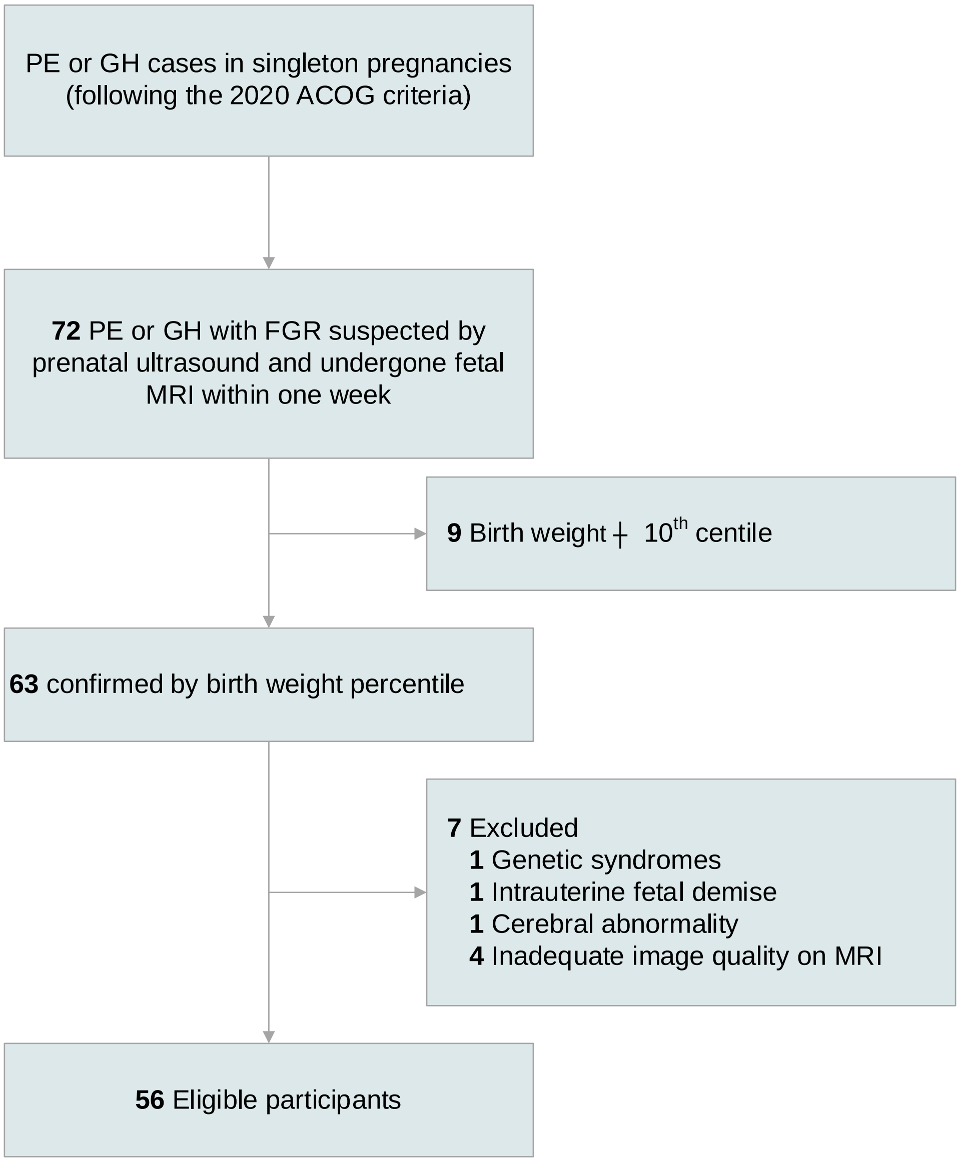

eFigure 1. A Flowchart Demonstrating the Selection of Study Subjects From the Enrolled Pregnant Women

Abbreviations: PE, preeclampsia; GH, gestational hypertension; FGR, fetal growth restriction; MRI, magnetic resonance imaging.

© 2022 Zheng W et al. *JAMA Network Open.*

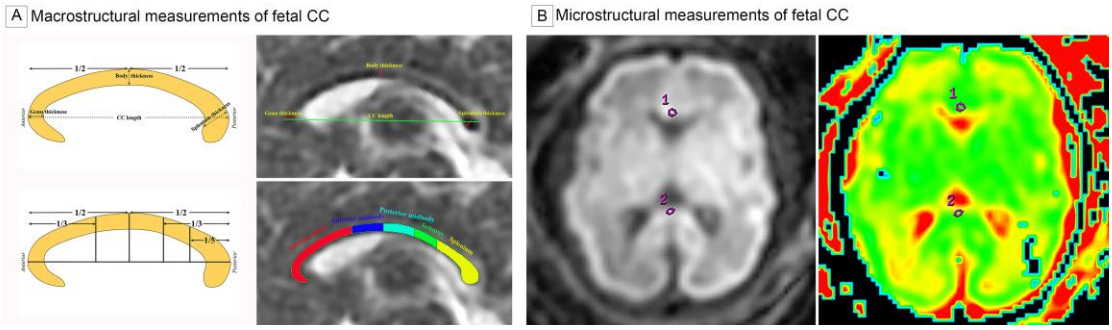

eFigure 2. The Macrostructural and Microstructural Measurements of the Fetal Corpus Callosum

(A) The linear and area measurements of the fetal corpus callosum are shown on the schematic illustration and midsagittal T_2WI of fetal MRI. CC length and CC thickness were measured. CC morphometry was assessed by measuring the total area and the area of a subdivision in five portions: anterior third, anterior midbody, posterior midbody, isthmus, and splenium region. **(B)** ADC values were measured in two regions of interests including the anterior third region (ROI 1) and splenium region (ROI 2) of the CC.

Abbreviations: CC, corpus callosum; ROI, regions of interest; MRI, magnetic resonance imaging.

© 2022 Zheng W et al. *JAMA Network Open.*

Parameters of fetal brain	N	ICCs [95% CI]	P value
BPD at MRI	42	0.993 [0.987-0.997]	< .001
OFD at MRI	42	0.996 [0.993-0.998]	< .001
CC length	42	0.890 [0.799-0.941]	< .001
Genu thickness	42	0.750 [0.582-0.857]	< .001
Body thickness	42	0.663 [0.089-0.861]	< .001
Splenium thickness	42	0.754 [0.366-0.891]	< .001
Anterior third	42	0.628 [0.128-0.832]	< .001
Anterior midbody	42	0.783 [0.629-0.877]	< .001
Posterior midbody	42	0.750 [0.524-0.868]	< .001
Isthmus	42	0.839 [0.637-0.922]	< .001
Splenium	42	0.708 [0.120-0.885]	< .001
Total area	42	0.812 [0.472-0.919]	< .001
ADC of anterior third	30	0.993 [0.985-0.997]	< .001
ADC of splenium	30	0.986 [0.972-0.994]	< .001

eTable 1. Intraclass Correlation Coefficients Results of Fetal Brain

Abbreviations: ICCs, intraclass correlation coefficients; BPD, biparietal diameter; OFD, occipitofrontal diameter; MRI, magnetic resonance imaging; CC, corpus callosum; ADC, apparent diffusion coefficient.

© 2022 Zheng W et al. *JAMA Network Open.*

Parameters of fetal brain	Less severe FGR (n=14)	Severe FGR (n=42)	P value
BPD at MRI, mean [SD], cm	8.32 [0.56]	7.87 [0.72]	.04
OFD at MRI, median [IQR], cm	9.76 [9.45-10.13]	9.21 [8.55-9.50]	.002
CI, mean [SD][b]	85.33 [6.41]	86.50 [4.73]	.47
CC length/CI, median [IQR], mm[b]	0.4412 [0.4257-0.4824]	0.4206 [0.4023-0.4439]	.03
Genu thickness/CI, median [IQR], mm[b]	0.0177 [0.0149-0.0210]	0.0202 [0.0152-0.0237]	.41
Body thickness/CI, mean [SD], mm[b]	0.0154 [0.0028]	0.0144 [0.0026]	.24
Splenium thickness/CI, median [IQR], mm[b]	0.0254 [0.0225-0.0312]	0.0274 [0.0203-0.0352]	.57
Anterior third/CI, mean [SD], mm^{2}[b]	0.4364 [0.0920]	0.3983 [0.0654]	.09
Anterior midbody/CI, mean [SD], mm^{2}[b]	0.1474 [0.0366]	0.1315 [0.0261]	.08
Posterior midbody/CI, mean [SD], mm^{2}[b]	0.1343 [0.0372]	0.1218 [0.0236]	.15
Isthmus/CI, mean [SD], mm^{2}[b]	0.1007 [0.0233]	0.0923 [0.0192]	.19
Splenium/CI, mean [SD], mm^{2}[b]	0.3488 [0.0784]	0.3036 [0.0666]	.03
Total area/CI, median [IQR], mm^{2}[b]	1.2028 [0.9637-1.3336]	1.0888 [0.9486-1.1436]	.08

eTable 2. Macrostructural Analysis of Fetal Corpus Callosum Between Less Severe FGR and Severe FGR Groups[a]

Abbreviations: FGR, fetal growth restriction; BPD, biparietal diameter; OFD, occipitofrontal diameter; MRI, magnetic resonance imaging; CC, corpus callosum; CI, cephalic index.

[a] The subjects of PE or GH with FGR were further classified as FGR and severe FGR groups.

[b] CI = BPD/OFD × 100.

© 2022 Zheng W et al. *JAMA Network Open.*

Parameters of fetal brain	GH-FGR (n=22)	PE-FGR (n=34)	P value
BPD at MRI, mean [SD], cm	8.22 [0.61]	7.83 [0.73]	.04
OFD at MRI, mean [SD], cm	9.53 [0.66]	9.10 [0.74]	.03
CI, mean [SD][b]	86.51 [6.36]	86.01 [4.31]	.73
CC length/CI, mean [SD], mm[b]	0.4439 [0.0657]	0.4243 [0.0311]	.20
Genu thickness/CI, median [IQR], mm[b]	0.0184 [0.0160-0.0243]	0.0189 [0.0146-0.0234]	.51
Body thickness/CI, mean [SD], mm[b]	0.0149 [0.0022]	0.0145 [0.0029]	.55
Splenium thickness/CI, median [IQR], mm[b]	0.0260 [0.0191-0.0312]	0.0283 [0.0228-0.0352]	.12
Anterior third/CI, mean [SD], mm^2[b]	0.4247 [0.0926]	0.3968 [0.0578]	.22
Anterior midbody/CI, mean [SD], mm^2[b]	0.1388 [0.0371]	0.1333 [0.0238]	.54
Posterior midbody/CI, mean [SD], mm^2[b]	0.1295 [0.0371]	0.1219 [0.0198]	.38
Isthmus/CI, mean [SD], mm^2[b]	0.0958 [0.0238]	0.0935 [0.0182]	.69
Splenium/CI, mean [SD], mm^2[b]	0.3240 [0.0784]	0.3090 [0.0640]	.44
Total area/CI, median [IQR], mm^2[b]	1.1142 [0.9459-1.2653]	1.0888 [0.9654-1.1344]	.23

eTable 3. Macrostructural Analysis of Fetal Corpus Callosum Between GH-FGR and PE-FGR Groups[a]

Abbreviations: PE, preeclampsia; GH, gestational hypertension; FGR, fetal growth restriction; BPD, biparietal diameter; OFD, occipitofrontal diameter; MRI, magnetic resonance imaging; CC, corpus callosum; CI, cephalic index.

[a] The subjects of PE or GH with FGR were further classified as GH-FGR and PE-FGR groups.

[b] CI = BPD/OFD × 100.

© 2022 Zheng W et al. *JAMA Network Open.*

Parameters of CC DWI	Less severe FGR (n=13)	Severe FGR (n=27)	P value
ADC of Anterior third, mean [SD], × 10^{-3} mm^2/s	1.46 [0.19]	1.50 [0.14]	.52
ADC of splenium, mean [SD], × 10^{-3} mm^2/s	1.52 [0.16]	1.46 [0.10]	.25

eTable 4. Microstructural Analysis of Fetal Corpus Callosum Between Less Severe FGR and Severe FGR Groups[a]

Abbreviations: PE, preeclampsia; GH, gestational hypertension; FGR, fetal growth restriction; CC, corpus callosum; DWI, diffusion weighted imaging; ADC, apparent diffusion coefficient.

[a] The subjects of PE or GH with FGR were further classified as FGR and severe FGR groups.

© 2022 Zheng W et al. *JAMA Network Open.*

Parameters of CC DWI	GH-FGR (n=16)	PE-FGR (n=24)	P value
ADC of Anterior third, mean [SD], × 10^{-3} mm^2/s	1.50 [0.16]	1.48 [0.16]	.70
ADC of splenium, mean [SD], × 10^{-3} mm^2/s	1.50 [0.15]	1.47 [0.11]	.38

eTable 5. Microstructural Analysis of Fetal Corpus Callosum Between GH-FGR and PE-FGR Groups[a]

Abbreviations: PE, preeclampsia; GH, gestational hypertension; FGR, fetal growth restriction; CC, corpus callosum; DWI, diffusion weighted imaging; ADC, apparent diffusion coefficient.

[a] The subjects of PE or GH with FGR were further classified as GH-FGR and PE-FGR groups.

© 2022 Zheng W et al. *JAMA Network Open.*
